# IL-4 Deficiency Decreases Mortality but Increases Severity of Arthritis in Experimental Group B *Streptococcus* Infection

**DOI:** 10.1155/2009/394021

**Published:** 2009-07-07

**Authors:** Luciana Tissi, Francesco Bistoni, Manuela Puliti

**Affiliations:** Microbiology Section, Department of Experimental Medicine and Biochemical Sciences, University of Perugia, Via del Giochetto, 06122 Perugia, Italy

## Abstract

IL-4 is an anti-inflammatory cytokine that inhibits the onset and severity in different experimental arthritis models. Group B streptococci (GBS) have been recognized as an ever-growing cause of serious invasive infections in nonpregnant adults. Septic arthritis is a clinical manifestation of GBS infection. To investigate the role of IL-4 in experimental GBS infection, IL-4 deficient or competent mice were inoculated with 1 × 10^7^ GBS/mouse. Mortality, appearance of arthritis, GBS growth in the organs, and local and systemic cytokine and chemokine production were examined. IL-4−/− mice showed lower mortality rates but increased severity of arthritis and exhibited a lower microbial load in blood, kidneys, and joints than wt mice. Increased local levels of IL-1 *β*, IL-6, TNF-*α*, MIP-1*α*, and MIP-2 accompanied the more severe arthritis in IL-4−/− mice. Our results suggest a detrimental role of IL-4 in GBS sepsis, whereas it plays a beneficial effect on GBS-induced arthritis.

## 1. Introduction

Group B streptococci (GBS) have long been known as a leading cause of life-threatening infection in neonates, young infants, and pregnant women [1–3]. Recently these microorganisms have been recognized as an ever-growing cause of serious invasive infections in nonpregnant adults [4–6]. GBS is responsible for a wide spectrum of clinical manifestations [5–9]. The most common entities include primary bacteraemia, skin and soft tissue infections, urinary tract infections, and pneumonia. Other relevant conditions are endocarditis, intravascular device infections, meningitis, peritonitis, endoophthalmitis and osteoarticular infections. Bacterial arthritis is a rapidly progressive and highly destructive joint disease in humans with an incidence rate ranging from 0.034% to 0.13% [10]. The typical case of this disease is characterized by high fever and a red, warm, and swollen joint, in 80% to 90% of cases displaying a monoarticular pattern. Patients with primary or secondary immunodeficiencies as well as those who have passed through prosthetic joint surgery display enhanced susceptibility to septic arthritis [10–12]. Septic GBS arthritis in nonpregnant adults was considered extremely rare [13, 14] until the early 1980s, when two independent studies stressed that its incidence seemed to be increasing [15, 16]. This trend has been confirmed by recent studies in which GBS account for 7% to 10% of all diagnosed cases of bacterial arthritis [17, 18]. Interestingly, GBS were typically associated with involvement of multiple joints in patients with diabetes mellitus or cancer [17]. 

We have previously described an experimental mouse model of type IV GBS infection with clinical features that closely resemble infection in humans [19, 20]. Mice given a single intravenous dose of GBS develop clinical signs of arthritis within 48 hours. Appearance and severity of GBS arthritis are the by-product of a multifactorial process. Viability and number of microorganisms injected and bacterial factors (i.e., presence and amount of capsule, amount of sialic acid in the capsular polysaccharide, and *β*-hemolysin production) have been shown to influence the development of articular lesions [21, 22]. Nevertheless, in the pathogenesis of GBS arthritis a crucial role is played by inflammatory cells (granulocytes and monocytes) that reach the joints [23, 24] and by the production of proinflammatory cytokines, including interleukin-6 (IL-6) IL-1*β* and tumor necrosis factor-*α* (TNF-*α*) [25]. 

Antiinflammatory cytokines such as IL-4 are known to regulate inflammation. Many of the IL-4 regulatory effects are directed at modulating macrophage activity. IL-4 suppresses proinflammatory cytokines (TNF-*α* IL-1*β*, and IL-12) [26, 27] and chemokines (interferon-*γ*-inducible protein 10 and macrophage migration inhibitory factor) [28, 29]. The antiinflammatory activity of IL-4 was assessed in vivo in several models of arthritis. It has been demonstrated that systemic administration of IL-4 effectively suppresses disease in proteoglycan-induced arthritis (PGIA) and collagen-induced arthritis (CIA) [30, 31]. Treatment with IL-4 or local expression of IL-4 in CIA prevents cartilage and bone destruction [32, 33]. 

The aim of our study was to assess the role of IL-4 in our septic arthritis model, both regarding development of the arthritic process and host susceptibility to bacteria. For this purpose, we used Balb/c wild-type mice and Balb/c mice with disrupted IL-4 gene.

## 2. Materials and Methods

### 2.1. Mice

Homozygotous IL-4−/− mice, generated as described elsewhere [34], have been kindly provided by Prof. Luigina Romani, University of Perugia, Perugia, Italy. Control wt Balb/c mice were purchased from Charles River Breeding Laboratories (Calco, Italy). Eight-to-ten week old mice were used for experimental procedures. The animals were maintained and used under strict ethical conditions according to recommendations of European Union's Directive no. 86/609/EEC.

### 2.2. Microorganisms

Type IV GBS, ATCC reference strain GBS 3139 (CNCTC 1/82), supplied by J. Jelinkova (Prague, Czech Republic) was used throughout the study. For experimental infection, the microorganisms were grown overnight at 37°C in Todd-Hewitt broth (Oxoid Ltd., Basingstoke, Hampshire, England) and then washed and diluted in RPMI 1640 medium (GIBCO, Life Technologies, Milan, Italy). The inoculum size was estimated turbidimetrically, and viability counts were performed by plating on tryptic soy agar −5% sheep blood agar (blood agar), and overnight incubation under anaerobic conditions at 37°C. A bacterial suspension was prepared in RPMI 1640 medium. Mice were inoculated intravenously via the tail vein with 1 × 10^7^ GBS in a volume of 0.5 mL. Control mice were injected by the same route with 0.5 mL of RPMI 1640 medium.

### 2.3. Clinical Evaluation of Arthritis and Mortality

GBS-infected mice were evaluated for signs of arthritis and for mortality. Mortality was recorded at 24-hour intervals for 30 days. After challenge, mice were examined daily by two independent observers (L. T., M. P.) for 30 days to evaluate the presence of joint inflammation, and scores for arthritis severity (macroscopic score) were given as previously described [26, 35]. Arthritis was defined as visible erythema and/or swelling of at least one joint. Clinical severity of arthritis was graded on a scale of 0–3 for each paw, according to changes in erythema and swelling (0 = no change; 1 point = mild swelling and/or erythema; 2 points = moderate swelling and erythema; 3 points = marked swelling, erythema, and/or ankylosis). Thus, a mouse could have a maximum score of 12. The arthritis index (mean ± SD) was constructed by dividing the total score (cumulative value of all paws) by the number of animals used in each experimental group.

### 2.4. Histological Assessment

GBS-infected mice were examined 10 days after infection for histopathological features of arthritis. Arthritic hind paws (1 per mouse) were removed aseptically, fixed in formalin 10% v/v for 24 hours and then decalcified in trichloroacetic acid 5% v/v for 7 days, dehydrated, embedded in paraffin, sectioned at 3-4 *μ*m, and stained with hematoxylin and eosin. Samples were examined under blinded conditions. Tibia-tarseal, tarsus-metatarseal, and metatarsus-phalangeal joints were examined, and a histologic score was assigned to each joint based on the extent of infiltrate (presence of inflammatory cells in the subcutaneous and/or periarticular tissues), exudate (presence of inflammatory cells in the articular cavity), cartilage damage, bone erosion, and loss of joint architecture. Arthritis severity was classified as negative (no infiltrate), mild (minimal infiltrate), moderate (presence of infiltrate, minimal exudate, and integrity of joint architecture), and severe (presence of massive infiltrate/exudate, cartilage and bone erosion, and disrupted joint architecture).

### 2.5. GBS Growth in Blood, Kidneys, and Joints

Blood, kidney and joint bacterial load in GBS-infected mice was determined by colony-forming-units (CFU) evaluation at different times after inoculation. Blood samples were obtained by retroorbital sinus bleeding before the mice were killed. Ten-fold dilutions were prepared in RPMI 1640 medium, and 0.1 mL of each dilution was plated in triplicate on blood agar and incubated under anaerobic conditions for 24 hours. The number of CFU was determined, and the results were expressed as the number of CFU per mL of blood. Kidneys were aseptically removed and homogenized with 3 mL of sterile RPMI 1640. All wrist and ankle joints from each mouse were removed, weighed, and homogenized in toto in sterile RPMI 1640 medium (1 mL/100 mg of joint weight). After homogenization, all tissue samples were diluted and plated in triplicate on blood agar, and the results were expressed as the number of CFU per whole organ or per ml of joint homogenate.

### 2.6. Sample Preparation for Cytokine Assessment

Blood samples from the different experimental groups were obtained by retroorbital sinus bleeding at different times after infection before the mice were killed. Sera were stored at −80°C until analyzed. Joint tissues were prepared as previously described [25]. Briefly, all wrist and ankle joints from each mouse were removed and then homogenized in toto in 1 mL/100 mg joint weight of lysis medium (RPMI 1640 containing 2 mM phenylmethylsulfonyl fluoride and 1 *μ*g/mL final concentration of aprotinin, leupeptin, and pepstatin A). The homogenized tissues were then centrifuged at 2000 g for 10 minutes, and supernatants were sterilized using a Millipore filter (0.45 *μ*m) and stored at −80°C until analyzed.

### 2.7. Cytokine Assays 

IL-6, IL-1*β*, TNF-*α*, macrophage inflammatory protein (MIP)-1*α*, and MIP-2 concentrations in the biological samples were measured with commercial ELISA kits purchased from R&D System (R&D Systems Inc., Minneapolis, Minn, USA) according to the manufacturer's recommendations. Results were expressed as picograms per mL of serum or supernatant from joint homogenates. The detection limits of the assays were 1.6 pg/mL for IL-6, 3 pg/mL for IL-1*β*, 1.5 pg/mL for MIP-1*α* 1.5 pg/mL for MIP-2, and 5.1 pg/mL for TNF-*α*.

### 2.8. Statistical Analysis

Differences in the arthritis index, number of CFU, and cytokine concentrations between the groups of mice were analyzed by Student's *t*-test. The log-rank test was used for data analyses of Kaplan-Meier survival curves. Incidence of arthritis and histologic data were analyzed by Fisher's exact test. Each experiment was repeated 3 times. *P*-values less than .05 were considered significant.

## 3. Results

### 3.1. IL-4 Deficiency Decreases Mortality But Increases Severity of GBS-Induced Arthritis

**Figure 1:**
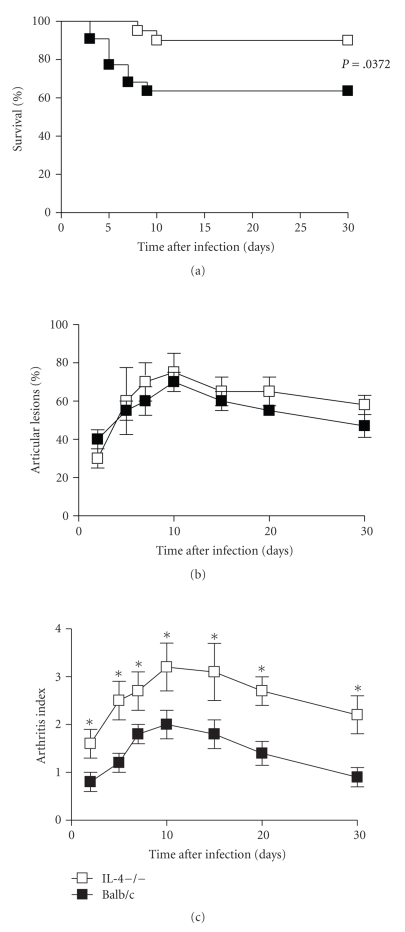
*Mortality rates, incidence, and severity of arthritis. * IL-4-deficient and wt mice were infected with 10^7^ GBS/mouse. Mortality (a), incidence (b), and severity (c) of arthritis were evaluated as detailed in materials and methods (see Section 2). Ten mice were used in each experimental group. (a) The data are the cumulative results of three separate experiments. (b) and (c) Data represent the mean ± SD of three separate experiments. Statistical differences between the experimental groups were marked with asterisk (*P* < .05).

Balb/c mice defective (IL-4−/−) or intact (wt) with respect to IL-4 gene were injected I.V. with 1 × 10^7^ GBS and the clinical course of disease was followed up to 30 days. The severity of the disease was monitored by assessing both survival rates and clinical score of arthritis. As shown in Figure 1(a) statistically significant (*P* = .0372) differences were observed in mortality rates between the two experimental groups. In fact, infection with 10^7^ GBS per mouse resulted in a 10% mortality in IL-4−/− mice, while 40% of wt mice had died at the end of observation period. The clinical signs of joint swelling were observed as early as 24 hours after injection of 1 × 10^7^ CFU of GBS in both groups of mice. Although similar frequency of articular lesions was observed in IL-4−/− and wt mice (Figure 1(b)), IL-4 deficient group had a more severe clinical arthritis in affected joints, compared with the control animals, resulting in a higher arthritis index (Figure 1(c)). Significant between-group differences were evident throughout the observation period, with maximum values reached at day 10 after infection of 3.2 ± 0.5 in IL-4−/− mice versus 2.0 ± 0.3 in wt animals.

### 3.2. Histopathological Findings

**Figure 2:**
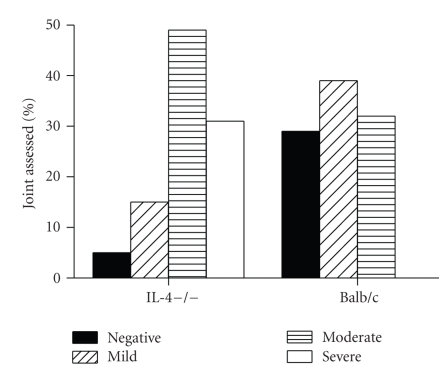
*Histopathological findings. * Histophatolgical severity of arthritis was assessed in IL-4−/− mice or controls 10 days after infection. Arthritis was defined negative, mild moderate, or severe as detailed in Materials and Methods (see Section 2). Cumulative results of two independent experiments are represented. For each experimental group, 5 arthritic paws from IL-4−/− mice or control mice were removed, and 3 joints for each paw were assessed for histopathological score.

Arthritic paws were removed for histological examination at day 10 after infection with 1 × 10^7^ GBS per mouse and examined for histopathological features of arthritis. Five arthritic paws from both IL-4−/− mice and BAlb/c mice were removed, and 3 joints for each paw were assessed for histopathological score. Two separate experiments were performed and results were cumulated. As shown in Figure 2, none of the joints from control animals could be classified as severely affected. In this group, 39% of the joints were classified as mildly affected, 32% as moderately affected, and 29% did not show any sign of arthritis. In contrast, In IL4−/− mice only 5% of the joints were negative, while the majority of them were classified as moderately (49%) or severely (31%) affected.

### 3.3. Lower Bacterial Growth in IL-4−/− Mice

**Figure 3:**
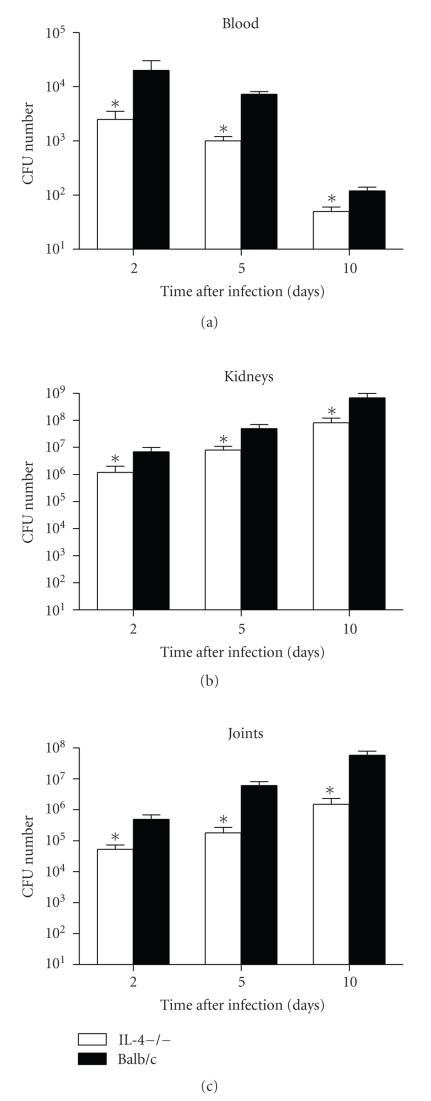
*Bacterial growth in blood, kidneys and joints. * IL-4-deficient and wt mice were infected with 1 × 107^6^ GBS/mouse, and blood and organs were removed and processed as detailed in materials and methods (see Section 2). The values represent the mean ± SD of three separate experiments each consisting of 20 mice per experimental group. Three mice per group were sacrificed at each time point. Results are expressed as number of CFU per ml of blood, per whole organ, or per ml of joint homogenate. Statistical differences between the experimental groups were marked with asterisk (*P* < .05).

In vivo GBS growth was assessed 2, 5, and 10 days after GBS infection by quantitative monitoring of bactaeremia and bacterial growth in kidneys and joints of IL-4−/− or wt mice. As shown in Figure 3, a progressive clearance of bacteria was observed in the blood of wt as well as IL-4−/− animals. However, it is noteworthy that a significantly (*P* < .05) lower number of GBS were recovered in the bloodstream of IL-4−/− mice in comparison to controls, as soon as two days after infection. Such a significant difference was still evident in the following days. A lower streptococcal growth was also observed in the kidneys from IL-4−/− mice with respect to controls. Ten-fold between-group differences in the number of GBS recovered were found at all the time points assessed. Finally, as already assessed in the kidneys and in the blood, bacterial burden in the joints of IL-4−/− mice was significantly lower than in controls, as soon as 2 days after infection. A progressive bacterial growth was then observed in the joints of both experimental groups, although a significantly lower bacterial load was still present in IL-4−/− mice.

### 3.4. IL-4 Deficiency Gives Rise to Upregulation of Local Cytokine and Monokine Production

**Figure 4:**
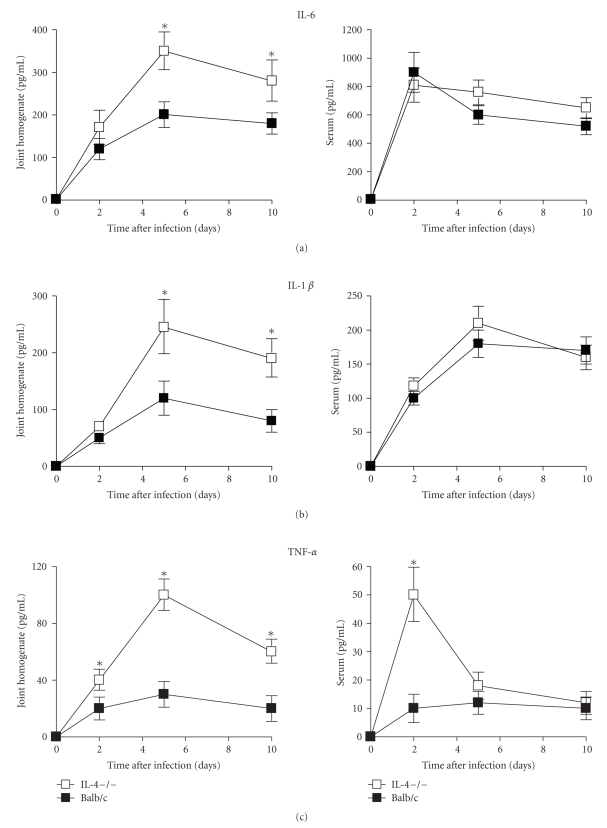
*Cytokine production in joints, and sera*. IL-4-deficient or wt mice infected with 1 × 10^7^ GBS/mouse. Supernatants from joint homogenates, or blood samples were collected at the indicated times after infection and assayed for TNF-*α*, IL-6, and IL-1*β* by ELISA as detailed in materials and methods (see Section 2). Values are the mean ± SD of three separate experiments each consisting of 20 mice per experimental group. Three mice per group were sacrificed at each time point. Statistical differences between the experimental groups were marked with asterisk (*P* < .05).

**Figure 5:**
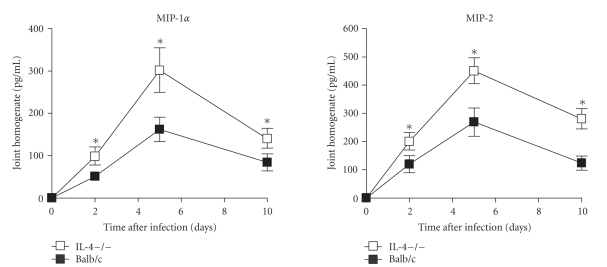
*Chemokine production in joints*. IL-4-deficient or wt mice infected with 1 × 10^7^ GBS/mouse. Supernatants from joint homogenates were collected at the indicated times after infection and assayed for Mip-1*α* and MIP-2 by ELISA as detailed in materials and methods (see Section 2). Values are the mean ± SD of three separate experiments each consisting of 20 mice per experimental group. Three mice per group were sacrificed at each time point. Statistical differences between the experimental groups were marked with asterisk (*P* < .05).

The role played by cytokines and monokines on the pathogenesis of GBS sepsis and arthritis is well-defined [24, 25]. Thus, local and systemic levels of IL-6, IL1*β*, TNF-*α*, MIP-1*α*, and MIP-2 were compared between IL-4−/− and control mice at 2, 5, and 10 days after infection. As depicted in Figure 4, higher levels of proinflammatory cytokines (IL-6, IL1*β* and TNF-*α*) were detected in the joints of IL-4−/− mice than in controls. At day 2 after infection significantly higher levels were evident only with regard to TNF-*α* production, but at the other time points assessed differences became statistically significant also for IL-6 and IL-1*β* production. On the contrary, serum levels of proinflammatory cytokines were similar in both groups, except for TNF-*α* production which was significantly higher in IL-4−/− mice with respect to controls (52 ± 10 versus 11 ± 5 pg/mL of serum) at day 2 after infection. A similar trend was observed for MIP-2 and MIP-1 production in both groups of mice, with peak values reached at day 5 after infection. Again, significantly higher levels of both chemokines were detected in the joints of IL-4−/− mice than in wt animals at all the time points assessed (Figure 5). Differently from the joints, no significant differences were observed between the two experimental groups with respect to systemic production of MIP-1*α* and MIP-2 (data not shown). 

## 4. Discussion

The murine model of GBS infection has proven to be beneficial in elucidating bacterial and host factors responsible for GBS arthritis [19–25]. Our previous studies pointed out a major role for proinflammatory cytokines such as of IL-6, IL-1*β*, IL-18, and TNF-*α* in the pathogenesis of GBS arthritis [25, 36]. In this study, by using deficient mice, we found that the antiinflammatory cytokine IL-4 has a relevant influence on GBS-induced sepsis and septic arthritis. The first effect of IL-4 absence on the course of GBS infection is the decrease of mortality rates. This result is in line with a paper by Hultgren et al., who described a beneficial effect of IL-4 lack in an experimental murine model of *Staphylococcus aureus * infection [37]. In this paper, the diminished mortality rates observed in IL-4−/− mice were associated to an increased systemic bacterial clearance, attributable to an enhanced intracellular killing capacity mediated by phagocytic cells. It has been demonstrated that IL-4 acts on phagocytes by suppression of oxidative bursts and intracellular killing of *Leishmania * [38], and inhibits peritoneal and splenic macrophage killing of *Candida albicans * [39]. Actually, in our experimental model a lower number of GBS were recovered both from blood and kidneys of IL-4−/− mice compared to controls, thus suggesting that IL-4 exerts an inhibitory effect also on GBS killing by phagocytes. 

Lack of IL-4 also results in a worsening of articular lesions. This was apparently in contrast with the lower bacterial load found in the joints of IL-4−/− mice with respect to controls. However, it must be considered that severity of GBS arthritis is not exclusively linked to the amount of bacteria present in the joints, but is also dictated by other parameters, including proinflammatory cytokine production and the extent of inflammatory infiltrate/exudates in the articular tissues. The recruitment and activation of inflammatory cells at infection sites is a complex and dynamic process that involves the coordinated expression of both pro- and antiinflammatory mediators. Indeed, although an inflammatory response is essential to clear pathogens from the site of infection, a prolonged inflammatory response might lead to a detrimental outcome [35]. In this respect, a beneficial effect of IL-4 has been demonstrated in many experimental model of arthritis. Administration of exogenous IL-4 resulted in suppression of CIA [30], while, in the same experimental model, administration of anti-IL4 monoclonal antibodies augmented both incidence and severity of arthritis [40]. Daily treatment with IL-4 was reported to reduce all disease parameters in a streptococcal cell wall (SCW) rat arthritis model [41]. In the PGIA model, arthritis was prevented by IL-4 treatment [31] and exacerbated in IL-4−/− mice [27]. In these mice, the rapid induction of arthritis parallels in vivo increase of proinflammatory cytokine and chemokine secretion. It is well known that proinflammatory cytokines and chemokines play a critical role in the pathogenesis of arthritis. Resident synovial macrophages and fibroblasts as well as infiltrate cells produce proiflammatory cytokines, such as TNF-*α*, IL-1, IL-6, IL-15, and IL-17 that drive disease pathogenesis [42, 43]. These cytokines initiate a cascade of events that induce cartilage-degrading enzymes, such as metalloproteases, and that stimulate osteoclastogenesis and angiogenesis [44–46]. Recruitment of cells to the synovial tissues involves chemokines, such as MIP-1*α* responsible for recruitment of mononuclear cells and MIP-2 that acts on neutrophils. All these inflammatory cells contribute to articular damage by cytokine and proteolytic enzyme production [11]. In our model, despite the lower number of GBS in the joints of IL-4−/− mice, all the other parameters leading to an exacerbation of articular lesions are manifested. In fact, worsening of joint disease was associated with a marked increase of local proinflammatory cytokines (IL-6, IL-1*β*, and TNF-*α*) responsible for articular damage, together with sustained levels of monokines and a consequent enhanced inflammatory cell influx.

In conclusion, we propose that IL-4 plays a dual role in the immune response to GBS infection. By one side, by inhibiting the intracellular killing of phagocytes, IL-4 may cause a more severe systemic infection and, consequently, higher mortality rates. By the other side, by downregulating local levels of proinflammatory cytokines and chemokines, it appears to positively influence the progression of GBS arthritis.
